# Role of the boundary in feather bud formation on one-dimensional bioengineered skin

**DOI:** 10.1063/1.4989414

**Published:** 2018-02-13

**Authors:** Kentaro Ishida, Toshiyuki Mitsui

**Affiliations:** Department of Physics and Mathematics, College of Science and Engineering, Aoyama Gakuin University, Kanagawa 252-5258, Japan

## Abstract

The role of a boundary in pattern formation from a homogenous state in Turing's reaction–diffusion equations is important, particularly when the domain size is comparable to the pattern scale. Such experimental conditions may be achieved for *in vitro* regeneration of ectodermal appendages such as feathers, via reconstruction of embryonic single cells. This procedure can eliminate a predefined genetic map, such as the midline of chick feather bud formation, leaving uniformly distributed identical cells as a bioengineered skin. Here, the self-organizing nature of multiple feather bud formation was examined in bioengineered 1D-skin samples. Primal formation of feather buds occurred at a fixed length from the skin edge. This formation was numerically recapitulated by a standard two-component reaction-diffusion model, suggesting that the boundary effect caused this observation. The proper boundary conditions were nonstandard, either mixed Dirichlet–Neumann or partial-flux. In addition, the model implies imperfect or hindered bud formation as well as nearly equal distances between buds. In contrast, experimental observations indicated that the skin curvature, which was not included in our model, also strongly affected bud formation. Thus, bioengineered skin may provide an ideal template for modeling a self-organized process from a homogenous state. This study will examine the possible diffusion activities of activator or inhibitor molecular candidates and mechanical activities during cell aggregation, which will advance our understanding of skin appendage regeneration from pluripotent or embryonic stem cells.

## INTRODUCTION

Turing's model of two reaction–diffusion equations has been applied to a wide variety of patterns in living systems. This mathematical model for simplifying complex phenomena has been successfully employed to evaluate various patterns and their formation while predicting the physical properties of various species, i.e., an activator and an inhibitor, in the models.^1,2^ Recent experimental studies of pattern formation have led to the identification of a number of molecular candidates for activator/inhibitor species and their underlying molecular mechanisms.^3,4^ This advance has led to the formulation of additional differential equations with nonhomogeneous initial conditions to generate the asymmetric order of multiple-structure formation in various complex patterns such as teeth, scales, feathers, hair, and other skin appendages during embryogenesis.^5–11^ Such complexities are likely predefined genetically and allocated appropriately on the segmented skin of each animal species.

In contrast, recent progress in the field of regenerative medicine, such as in tissue engineering, has facilitated simplification of Turing's model.^12^ In such a tissue sample, a self-organized pattern formed from a homogeneous state. This process was attributed to the fact that bioengineered tissue is composed of assembled identical cells such as induced pluripotent stem cells or embryonic stem cells (ESCs).^13,14^ Therefore, reaction–diffusion equations for simulating experimentally observed patterns may be simplified by using homogeneous initial conditions. This homogeneity increases the sensitivity to the domain shape and boundary edge. This situation becomes particularly important when the simulation domain size is examined at the characteristic scale of the pattern. This information can be applied experimentally in *in vitro* assays for regenerating teeth, hair, or feathers, which involve the assembly of induced pluripotent stem cells or ESCs.^12,15–19^

Similar state-of-the-art bioengineering methods have been developed to generate *in vitro* skin appendages by assembling single cells from both the embryonic epithelium and mesenchyme and then combining the epithelium and mesenchyme as a bilayer. Teeth,^10,20,21^ hair,^22–24^ and feathers^25^ have been successfully generated with a nearly uniform size on such reconstructed bilayers, which are referred to as bioengineered skin. The skin shape is typically rectangular, on which a single appendage array forms.^18,23,25,26^ We previously found that rectangular bioengineered skins of chick embryos can generate several feather buds.^25^ This may provide sensitivity to the skin edge for bud formation, and the results can be used to investigate the role of boundary conditions (BCs) and validate simulation-based mathematical models.^27,28^

A similar reconstruction assay, dissociating and reassembling only the mesenchyme (not the epithelium), laid on a native 2D epithelium of chick skin was performed by Jiang *et al.*^29,30^ The bioengineered skins induced bud formation with nearly uniform spacing on the 2D skins rather than forming a hexagonal bud pattern starting from the midline, with a single bud row at the center appearing on a native chick skin. It is noteworthy that the reconstruction assay showed that bud formation is initiated by cell aggregation under the influence of mechanical tractability.^31^ However, this reconstruction assay leaves anterior–posterior (AP) asymmetry on the engineered skins as the buds are tilted in the same direction, presumably because the native epithelium contains positional information for AP asymmetry.^29,30,32–35^

To prepare completely homogeneous bioengineered skins, we attempted to dissociate both the epithelium and the mesenchyme into single cells before reconstructing them. We previously examined the expression patterns of morphogens in the generated buds in an array on rectangular, nearly 1D bioengineered skin samples on day 3.^25^ On the skins, there was no clear correlation between the number of buds and the skin length; particularly, the skin length was short.

Here, we evaluated the spatiotemporal development of feather bud patterns on near-1D bioengineered skins, particularly on which fewer than three buds could grow to focus on the skin length vs. bud number and the skin edge effects. Bud orientation was also examined to confirm elimination of the AP asymmetry. First, we determined the primary location of bud formation on the bioengineered skins. The locations were a constant distance from the skin edge. Second, we examined these primary locations numerically using a two-component Turing model under homogenous initial conditions.^2,7,36,37^ The simulation results with homogeneous BCs did not replicate the experimental results, while simulation with non-standard BCs either mixed Dirichlet–Neumann^27^ or partial-flux^38^ did. A time course of the simulation results revealed possible factors, resulting in limited or imperfect bud formation. Additionally, other factors, such as skin curvature, affected failed bud formation. By taking these factors, particularly those affecting failing formation, into account, the simple relationship between the skin length and the bud number was determined from the reaction diffusion model. In this model, we considered the physical properties such as the diffusion coefficient of the activator and the inhibitor as factors for cellular contractility of a 1D bioengineered skin. For developing the model as a biophysical model rather than a simple mathematical model, such as by including cell density, our results provide information related to geometrical transformation induced by cell proliferation and migration.

We examined the importance of the size and the shape, particularly the edge of bioengineered tissue formed from uniformly distributed homogeneous cells for regenerative medicine. As a remark, similar edge effects were also found recently using human ESCs *in vitro*.^39,40^ Such bioengineered tissue samples may provide ideal templates for studying or modeling a self-organized process for biophysical models,^2,41,42^ contributing to the advancement of regenerative medicine.

## RESULTS

### Feather bud formation on near-1D bioengineered skin samples

**FIG. 1.**
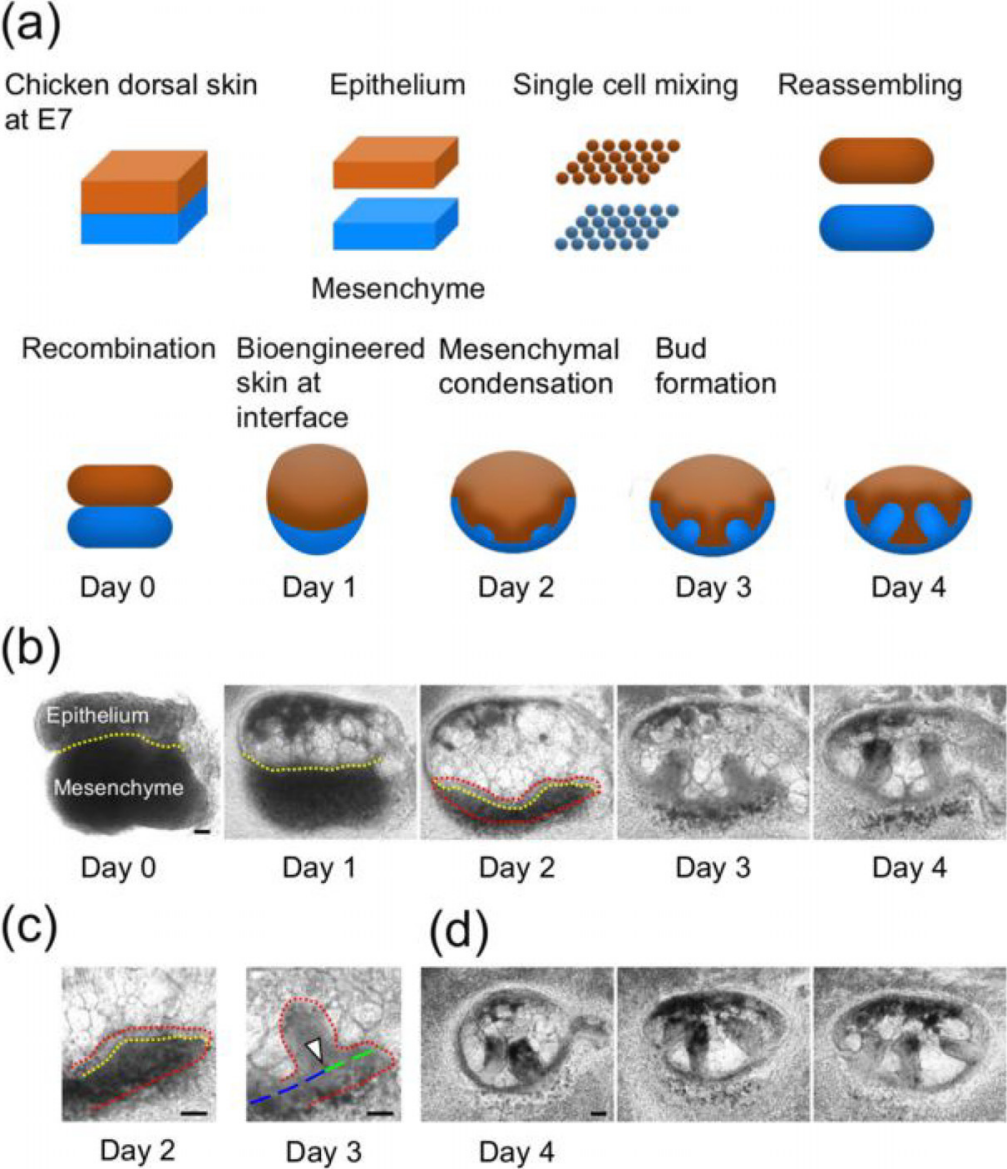
Formation of feather buds on bioengineered skin. (a) Schematic representation of skin fabrication, bud formation, and skin shape transformation. (b) Phase-contrast images of the development of two buds from days 0 to 4. Dotted yellow lines indicate the epithelium-mesenchyme interface. On day 2, dotted red lines indicate the whole skin, epithelium (top), and mesenchyme (bottom), and two projections were observed because of mesenchymal condensation. The projections developed into buds on day 3 and grew further on day 4. (c) High-magnification images on days 2 and 3. The uniform thickness of an epithelial layer was observed above the dotted yellow line on day 2. The arrow head points to the location of the bud on day 3. The dashed blue and green line indicates the baseline for measuring the locations of the bud on the skin. (d) Two- or three-bud skins on day 4. The tips of nearby buds came into contact, as the buds had grown perpendicularly to the curved skin layers. All scale bars in the phase-contrast images are 100 *μ*m.

**FIG. 2.**
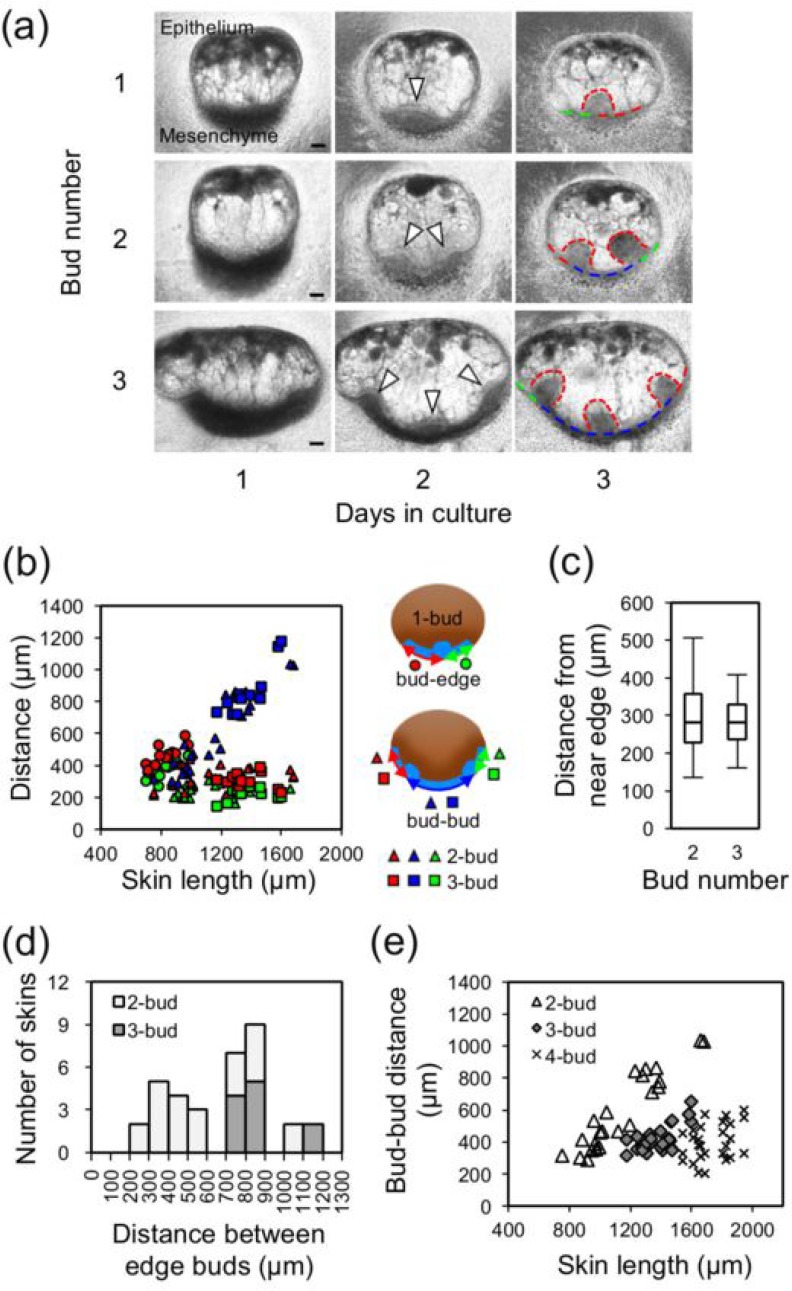
One, two, and three buds on bioengineered skins. (a) Phase-contrast images of the development of one, two, and three buds from days 1 to 3. Arrowheads indicate the locations of mesenchymal condensation on day 2. Dotted red lines indicate the outline of buds on day 3. Dashed lines indicate skin layers to measure bud locations, distances between near-edge-buds (blue), and from each edge (red and green). For edge-to-bud distances, red lines indicate longer distances. The skin lengths are 705, 959, and 1583 *μ*m for one, two, and three buds, respectively. All scale bars are 100 *μ*m. (b) Scatter plot of skin length versus bud locations on day 3. Schematics indicating the distances from the skin edge or between buds, with marker shapes and colors assigned. The edge-bud distances were approximately uniform regardless of the skin length on two- and three-bud skins. (c) Box plots representing the edge-bud distances for two- and three-bud skins, and the middle line represents the median, 280 and 281 *μ*m, respectively. (d) Histogram of the distances between near-edge buds on day 3. There was no clear threshold of the skin length between two and three buds. (e) Nearest neighbor bud-bud distances. Except for two-buds longer than 600 *μ*m, the distances were approximately 400 *μ*m.

The schematics in Fig. 1(a) show the method used to fabricate bioengineered skin. This approach involved bilayer formation using reassembled aggregates of epithelial and mesenchymal cells, followed by their combination, and feather bud formation initiated by mesenchymal condensation at the interface layer. As a representative example, microscopy images of the two bud formation sequences on bioengineered skin from day 0 to 4 are presented in Fig. 1(b). The details of the fabrication method and the study of developmental signals and morphogens of bioengineered buds are reported in our previous publication.^25^ We initially focused on morphological changes in the bioengineered skin samples. On day 0, the cylindrical epithelium was attached to the cylindrical mesenchyme, arranged in parallel orientation, on a plate in a gel drop [Fig. 1(a)]. The morphological features changed dramatically, particularly during the first 2 days of incubation, as shown schematically in Fig. 1(a); an experimentally observed image sequence is shown in Fig. 1(b). First, the cylindrical epithelium expanded by nearly 2-fold and transformed into an oval-shaped structure. In contrast, the cylinder shape of the mesenchyme appeared thinner and changed its length during epithelium expansion. As a result, the mesenchyme appeared as a U-shaped wrapping around the oval epithelium, thereby establishing an interface layer, which was considered the bioengineered skin. Thus, skin lengths also varied from 5% to 23% during the initial 3 days of incubation, primarily during the two days, after which the values stabilized. The height of the oval, which was perpendicular to the plane of microscopic imaging, was 334 ± 12 *μ*m, indicating that the oval was flattened, possibly by gravity. We previously explored the origin of this shape transformation, including epithelium expansion. The void or cellular carcass fills the center of the swollen epithelium and leaves a healthy epithelium layer at the periphery of the oval shape.^25^ This shape transformation generated slightly curved bioengineered skin types, i.e., epithelium–mesenchyme bilayers, with the side view of bioengineered skin shown in Figs. 1(b) and 2(a). Because we focused on the formation of multiple buds (three or fewer), skin lengths <1700 *μ*m on day 3 are primarily discussed in this article.

During the initial stage of bud formation, mesenchymal condensation was clearly visible by day 2. Morphogen expression indicates that the bud location becomes visible after approximately 30 h.^25^ The high-magnification images in Fig. 1(c) depict condensation beneath the epithelial layer, followed by bud formation. The dotted yellow line in Fig. 1(c) (for day 2) indicates the boundary between the epithelium and the mesenchyme. The thickness of the epithelial layer was relatively uniform (28 ± 2 *μ*m), whereas that of the mesenchymal layer was 46–200 *μ*m, indicating maximal mesenchymal condensation at the center. The columnar growth of a feather bud at the maximal center is shown in Fig. 1(c) (for day 3). The center of the bud column (indicated by an arrow) was 199 *μ*m away from the skin edge (dashed green line). We have shown the substrate skin layer by the dashed green and the blue line to measure bud locations while ignoring the projection by the bud. The direction of columnar growth was perpendicular to the curved skin layer. The growth direction was nearly conserved even after day 4, although ∼75.4% (n = 46/61) of the oval epithelium decreased in volume after day 3. Representative columnar buds on day 4 are presented in Fig. 1(d). Because the substrate skin samples were curved, the tips of nearby buds came into contact by day 4. In contrast, no columnar buds were perpendicular to the layer of native skin. They became tilted toward the posterior on native skin, reflecting AP asymmetry by day 4.^34^ This AP asymmetry exists in previously described bioengineered skin formed by reassembling single mesenchymal cells attached to nondissociative native epithelial tissues, as tilted buds formed on the skin by day 4.^29,43^ This finding indicates that AP asymmetry, presumably preregistered in a native epithelium, was lost in our bioengineered skin samples after dissociation of the epithelium into single followed by reassembly.

The buds that developed on our bioengineered skin samples were transferred on day 3 onto a chorioallantoic membrane of an E8 embryo (which ensured ovolike culture conditions). These buds continued to grow after day 4 as columnar buds. After the development of blood vessels in the bioengineered skin samples via the substrate membrane, nearly half of these columnar buds exceeded 700 *μ*m in height toward the feather follicles for 7 days of incubation.^25^

### Preferential locations of bud formation on bioengineered skin

There was no clear-cut threshold between the bud number (particularly less than four) and bioengineered skin lengths, as reported previously.^25^ To examine how the bud number was determined on the bioengineered skin samples, we analyzed bud locations within 1D skins. Figure 2(a) shows representative examples of 1–3 bud formation sequences from days 1 to 3. The locations of mesenchymal condensation are indicated by arrowheads on day 2. The formed buds are outlined by red dotted lines. To collate the bud locations with the skin lengths, the distances between buds and skin edges with respect to skin lengths were plotted for one-, two-, and three-bud skin types, as presented in Fig. 2(b). For one-bud skin samples, the shorter distances to an edge were plotted as green circles, whereas the others are shown as red circles. Similarly, the distances from near-edge buds to the edge for two and three buds were plotted as triangles and squares, respectively. Shorter distances are marked in green, whereas the others are marked in red. To delineate an edge effect for bud formation, the distances between near-edge buds for two and three buds were plotted in blue. [See the phase contrast images on day 3 in Fig. 2(a) and schematics in the inset of Fig. 2(b)]. The locations of the center buds for three buds are shown in Fig. 4(b), as described below. Figure 2(e) presents bud-bud distances, nearest neighbor, for two-, three-, and four-buds, which shows that the plots for two-bud are the same as the blue triangles in Fig. 2(b).

This plot revealed nearly uniform distances from a near-edge bud to the edge regardless of the skin length, whereas the distances between near-edge buds marked in blue for two- and three-bud skin samples linearly increased with the skin length. As a reference, Fig. SI1 (supplementary material) shows a plot of skin length versus bud locations [Fig. 2(b)] for up to four-bud skins, which shows the same features. The box plot of distances from a near-edge bud to the nearest edge in Fig. 2(c) revealed similar median values of 280 and 281 *μ*m for two- and three-bud skin samples, respectively. This finding implies that the preferred locations for bud formation on the bioengineered skin are near the edge and then the third bud forms between the edge buds. There was no clear correlation between an edge bud to edge bud edge distance and the number of buds, namely, the center bud formation as the distance from 700 and 900 *μ*m contained either two or three buds as shown in Fig. 2(d). In Fig. 4(b), this appeared as the overlap of the skin length particularly from 1200 to 1400 *μ*m for two or three buds. The overlap for one or two buds was also seen from 800 to 1000 *μ*m. The overlap was also identified in a histogram of the skin length in Fig. 4(a). The skin length is the edge bud to edge bud distance added to a pair of near-edge buds to nearest edge distances. Notably, similar skin lengths for forming either two or three buds were observed with/without a center bud. This gave a factor of two between these bud-bud distances, revealed in Fig. 2(e) as the distances for two-bud (9 triangles) located approximately 800 *μ*m, while the others were approximately 400 *μ*m. Except these, bud-bud distances are 416 ± 89, 418 ± 88, and 406 ± 113 *μ*m (mean ± s.d.) for two-bud, three-bud, and four-bud, respectively.

### Numerical simulation of two-component reaction–diffusion equations with selected BCs

Two partial differential equations for activation and an inhibitor based on Turing's reaction–diffusion model^2^ were applied to determine the preferential locations of bud formation on our bioengineered skin. The domain was 1D-like and formation of only three or fewer buds occurred in an array limited by the skin length. The activator peaks considered the bud locations for these two-component equations although recent Turing's reaction–diffusion models contain three or more equations, including cell density directly related to bud formation, which is used to simulate more complex biological patterns, such as AP asymmetry, during embryogenesis.^8^ Our reconstructed experimental conditions were not bound by the constraints of genetic memory for site-specific expression, which requires nonhomogeneous initial conditions. It is presumably ideal to test a two-component equation of pattern formation using homogenous initial conditions, operative in a self-organized manner despite the limited domain shape and size. Because of the limitations, the activator profile tends to be sensitive to the BC near the boundary. Although the behavior of the activator near the boundary has been examined numerically using various BCs,^27,28^ we also performed numerical simulations on 2D domains similar to our 1D-like bioengineered skin samples. For BCs, zero-flux,^2^ partial-flux,^38^ and mixed Dirichlet–Neumann^27^ were tested to examine activator peak locations.

**FIG. 3.**
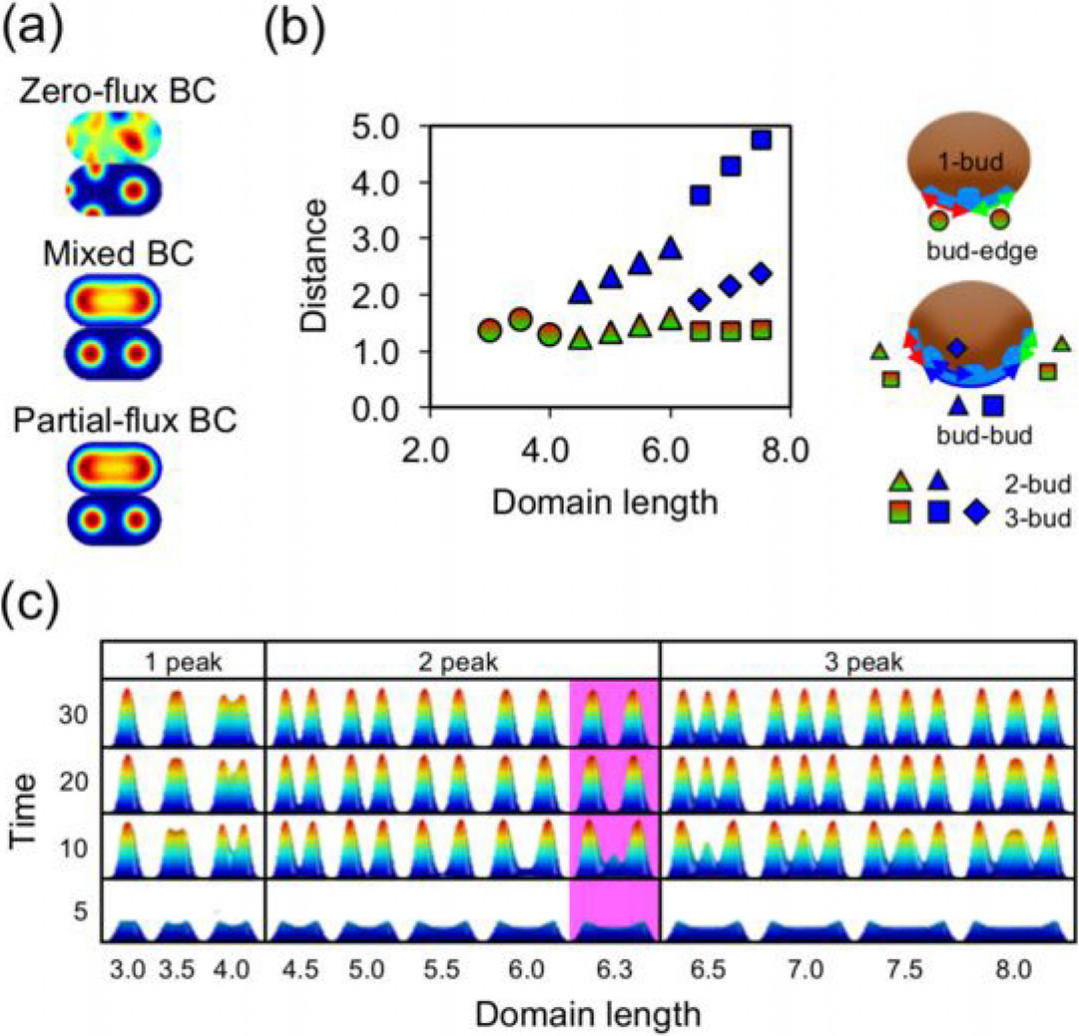
Simulation results of bud locations. (a) Activator profiles at 5 and 30 time units using various BCs. Zero-flux BC generated activator peaks at the boundary edge. Mixed and partial-flux BC generated activator peaks at a constant distance from the edge. (b) Scatter plot of the domain length versus activator peak locations at 30 time units. The inset schematics illustrate the distances for measuring bud locations. The marker shapes and colors are the same as those in Fig. 2(b) except for the edge-bud distances and bud-bud distances of 3-bud (rhombus). A pair of these distances was the same because of symmetricity. (c) Side view of activator profiles using mixed BC on selected domain lengths. Peaks were observed near the edge as early as 5 time units on the domain length units, 3.5–8.0. A pair of near-edge peaks developed on the domain length units, 4.5–8.0. Center peaks grew after the near-edge peaks were developed on the domain length units, 6.5–8.0. Notably, the small center peak at 10 time units on the 6.3 domain disappeared later. The apex of the center peak on the 8.0 domain was not as sharp as the others at 10 time units. Multimedia views: https://doi.org/10.1063/1.4989414.1
10.1063/1.4989414.1; https://doi.org/10.1063/1.4989414.2
10.1063/1.4989414.2; https://doi.org/10.1063/1.4989414.3
10.1063/1.4989414.3; https://doi.org/10.1063/1.4989414.4
10.1063/1.4989414.4; https://doi.org/10.1063/1.4989414.5
10.1063/1.4989414.5; https://doi.org/10.1063/1.4989414.6
10.1063/1.4989414.6; https://doi.org/10.1063/1.4989414.7
10.1063/1.4989414.7; https://doi.org/10.1063/1.4989414.8
10.1063/1.4989414.8; https://doi.org/10.1063/1.4989414.9
10.1063/1.4989414.9; https://doi.org/10.1063/1.4989414.10
10.1063/1.4989414.10; https://doi.org/10.1063/1.4989414.11
10.1063/1.4989414.11; https://doi.org/10.1063/1.4989414.12
10.1063/1.4989414.12

Zero-flux, known as Neumann BC, is the most typical BC applied to Turing's reaction–diffusion models. This is because species do not pass the boundary fits to most biological conditions. However, this BC with homogenous initial conditions generates maximum peaks for the activator at the boundary edge because the activator returning at the boundary self-enhances the activator as shown in Fig. 3(a). These peaks were not suitable for our experimental studies. The locations of activator peaks can be accurately determined using two types of BCs. The first pertains to partial-flux BC, as introduced by Painter^38^ based on Mou's experimental results obtained from embryonic skin cultures.^6^ This BC allows the activator to partially cross the boundary, while the inhibitor does not leave as Neumann, zero-flux BC. The other reflects a mixed BC. For example, the Dirichlet fixed concentration pertains to a zero concentration at the boundary for the activator and Neumann and zero-flux for the inhibitor.^27^ These exceptional BCs are known to reduce or eliminate the activator concentration at the boundary. In contrast, enhancement of the inhibitor at the boundary of zero-flux occurs at the boundary, which inhibits the activator. As a result, the activator peaks formed at a constant distance from the boundary for the 1D domain^27^ or the large 2D domain.^38^ We studied domains with similar shapes for the bioengineered skin for all BCs described above. These are shown in Fig. 3(a) for a domain of 5.5 length units and Fig. SI2 (supplementary material) for various lengths. As expected, the exceptional BC generated peak locations at approximately the same distance from the edge for all domain lengths. The duration of the development of activator peaks was also similar between these BCs, as shown in Fig. 3(c). At as early as 5 time units, a pair of peaks was observed near the edge on domains of at least 3.5 length units and subsequently formed corn peaks on domains of at least 4.5 length units. On the 4.0 domain, the near-edge peaks merged into a single corn peak by 50 time units. All other activator peaks showed stabilized shapes and locations by 30 time units; the relative locations are plotted in Fig. 3(c), and activator peaks with mixed BC are summarized in Fig. 3(b) (also see Multimedia view). Because of the near-edge buds at a constant distance from the boundary, the nearest bud-bud distance increased slightly with the domain length until the distance decreased after another bud formed between buds between domains 6.0 and 6.5 as shown in Fig. 3(c). This produced the sawtooth profile tracing of the blue triangles to the blue rhombuses of the bud-bud distances shown in Fig. 2(b). Apparently, the profile of these distances approximately fits our experimental observations, as shown in Fig. 2(b); the relevance and discrepancies are described in the Discussion section.

The other feature of these two BCs is that the third peak at the center grew after near-edge peaks formed on domains longer than 6.5 length units. The growth speed of the center peaks was slower for shorter skin lengths. At 10 time units, the center peaks were small on the 6.5 domain and broad on the 8.0 domain but nearly as sharp as the near-edge peaks on the 7.0 and 7.5 domains. We added the 6.3 domain, highlighted in Fig. 3(c), whose length was slightly shorter than 6.5 length units with three peaks formed. Remarkably, a small center peak, formed initially at 10 time units, decayed later. The details of simulation parameters such as the partial-flux ratio of the activator are shown in supplementary material Table 1.

## DISCUSSION

The results of our *in vitro* experiments indicate that the primal locations for feather bud formation were near the edges of 1D-like skins. In the simulations, this effect reflected the nature of the zero-flux boundary for the long-range inhibitor, which suppresses activator growth. At the same time, the activator must sink at the boundary. Similar bud formation at a constant distance from the boundary edge has been observed experimentally on fragments of 2D native skin from an embryonic mouse *in vitro*^6^ and detected numerically using a partial-flux BC for the activator.^38^ In both native and bioengineered skin samples, the skin edge is likely not a perfect boundary wall for morphogens corresponding to the activator; this prevents mesenchymal cells from condensing directly on the edge. In contrast, the boundary may function as a wall for morphogens corresponding to the inhibitor because a zero-flux BC for the inhibitor is suitable for suppressing activator growth. These features of the morphogen boundary fix the location of feather bud formation.^27^ In addition to the edge effect, a simple reaction diffusion model predicted the equidistance between neighbor buds in a symmetric manner. This was also observed as the bud-bud distance was fairly equal at ∼400 *μ*m on skins up to four-bud, as shown in Fig. 2(e), although there were a few exceptions as shown in Fig. 2(e).

We next attempted to predict the edge effect without applying the BCs to a two-component equation for pattern formation. If the constant *k*_2_, sensitivity to suppress the activator, in Eq. (1) changes to the function of space, ex. *k*_2_(*x*) = *k*_2_′*tanh* (*x*–*L*), a constant value, *L,* fixes the location of bud formation.^7,44^ Such spatial variations in parameters have been applied to a two-component equation of the activator and the inhibitor in order to simulate complex patterning during embryonic development, assuming the spatial heterogeneity of cells.^27^ However, such spatial heterogeneity is not suitable to predict our experimental results because our bioengineered skin was spatially homogeneous.

Several molecular candidates for morphogens have been suggested as activators or inhibitors, and their regulatory pathways in feather bud formation are summarized in a previous review.^11^ The bone morphogenetic protein (BMP) family,^45,46^ such as BMP2 and BMP12, but not BMP7,^47^ functions as inhibitors, while fibroblast growth factors (FGFs)^48^ and Wnt/β-catenin^49,50^ act as activators. These molecules typically have very similar diffusion coefficients of approximately 100 *μ*m^2^/s and free diffusion coefficients (*D*) because they are 20–30-kDa molecules with similar protein structures.^51^ This does not generate a Turing's pattern where *D* for the inhibitor must be 20–100-fold larger than the activator for a long-range pattern in a homogenous field.^2,36^ The difference in *D* appears to be induced by the gap junction^52^ and extra-cellular matrix.^53–55^ These substrates limit or bind the morphogens and decrease *D*. Recently, *D* for BMP was measured experimentally to be 15 *μ*m^2^/s in *Xenopus*,^56^ 18 *μ*m^2^/s (Lefty2, the same family of BMP) in zebrafish, and 21 *μ*m^2^/s (Dpp, a homologue of BMP) in the developing Drosophila wing disc.^57^ In contrast, for the activator, *D* of Wg, a homologue of Wnt, is 0.05 *μ*m^2^/s in the developing fly wing^58^ and *D* of FGF8 is 4 *μ*m^2^/s in zebrafish embryo.^59^ Typical lengths for a chick bud pattern were 400 *μ*m for between buds and 280 *μ*m for the skin edge to the near-edge bud. The ratio between these numbers matched our simulation results, 2.2 between buds to 1.4 edge-bud as shown in Fig. 3(b). The diffusion coefficients were 38 *μ*m^2^/s for the inhibitor and 0.76 *μ*m^2^/s for the activator, as estimated from the simulation parameters. These *D* values are very similar to the values found in previous studies, and the diffusion distance for the slower activator was estimated to be approximately 360 *μ*m in 24 h (√2Dt in one direction). However, to our knowledge, no studies have detected 20–100-fold differences in *D* values between BMP as an inhibitor and FGF as an activator in a developing biological tissue. The other indication predicted by the simulation was the behavior of morphogens at the bioengineered skin edge, which transported the activator out while restricting the inhibitor. These morphogens likely diffuse through the cell membrane in skin tissue as patterns emerge.^38^ Therefore, activators may leave across the skin edge. However, the mechanism of BMP bouncing off at the tissue edge is unknown although a similar phenomenon in the enhancement of BMP was observed at the boundary of the imaginal disc of the fly wing, in which BMP expression was enhanced, as well as at the tip of the limb bud and fingers.^60,61^

In addition to the Turing model, several models have been introduced to describe pattern formation in biological systems.^62–64^ The goal is to not only to avoid diffusion coefficients D_inhibitor_ ≫ D_activator_ but also to include mechanical interactions as environmental factors. If mechanical stimuli can modify the bud pattern, these models will be the first-choice for reflecting a modified pattern, where cellular contractility is considered as an activator and substrate stiffness as an inhibitor in the Turing model.^31^

**FIG. 4.**
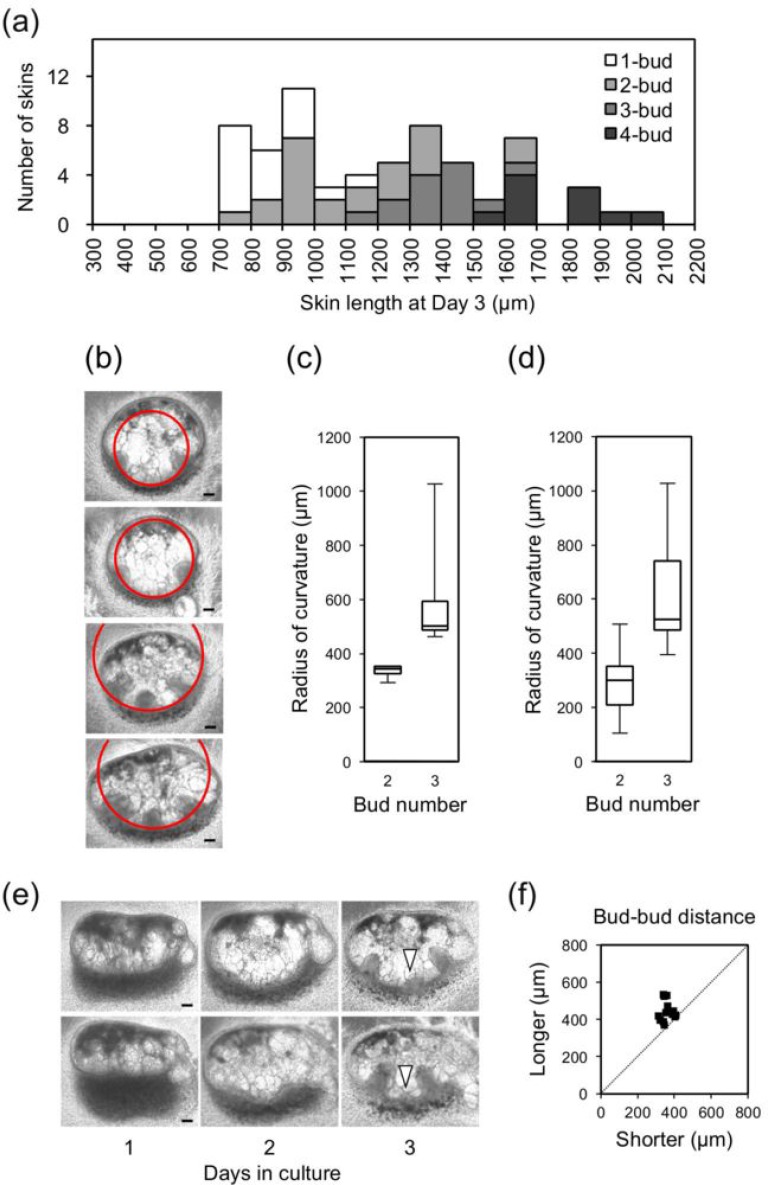
(a) Histogram of bioengineered skin lengths on day 3. One- and two-bud skins mainly showed lengths of 800–1000 *μ*m, while two- and three-bud skins showed lengths of 1200–1400 *μ*m. (b) Representative two- or three-bud skins of 1200–1400 *μ*m on day 3. Red circles denote the curvature of the skins near the center and radius of the curvatures, which were 353, 353, 495, and 508 *μ*m from the top to the bottom. Scale bars: 100 *μ*m. (c). (d) Box plots of the skin curvature of the skins at 1200–1400 *μ*m (c) and all (d). Particularly, (c) indicates the higher curvature preventing the bud from forming at the center. (e) Short buds at the center of skin length of 1394 *μ*m and bud-bud length of 833 *μ*m (upper) and 1158 *μ*m and bud-bud length of 568 *μ*m (lower). (f) Position of the center bud on three-bud skins. *x* axis indicates the shorter length from the center bud to near-edge buds.

There were some discrepancies between the experimental observations and numerical simulations. As described in the Results section, the bud number overlapped with the skin length as shown in Fig. 4(a). This indicates the influence of factors beyond our simple two-component Turing model that recaptures bud formation. Eight two-bud skins longer than 1200 *μ*m, which were sufficient to form three buds, were all missing a center bud as shown in Fig. 2(b), as the bud-bud distances were nearly 800 *μ*m; these values are two-fold greater than other values for similar skin lengths, as shown in Fig. 2(e). Observation of skins, particularly those of approximately 1300 *μ*m, revealed a correlation between the skin curvature near the center and center bud formation. Figure 4(b) shows that the radii of the curvatures were 338, 353, 495, and 508 *μ*m from top to bottom. The center bud formed when the radius of the curvature was more than 400 *μ*m. The curvature vs bud number for all two- and three-skins is also plotted in Fig. 4(d) and again revealed a correlation. This indicates that the skin curvature may suppress center bud growth. The skin surface at locations with a higher curvature generally generates appendages.^65,66^ In this study, the polarity of the skin curvature was negative. This may have the opposite effect, as dents function as “mechanical” inhibitors.^67^

Similar to the mechanical effects, in the recent report by Shyer *et al.*, it was found that the cellular self-organization and mechanosensation initiated the follicle and pattern formation.^31^ This observation indicates that the mechanical instability is accompanied by reaction-diffusion instability, where the activator and the inhibitor are mechanical cellular contractility and substrate stiffness, respectively. On the developed bioengineered skin, the contractility and stiffness likely differ near the edge as compared to the center because of the 1-D mesenchyme geometry attached to the oval shape epithelium. This may generate buds at a constant distance from the skin edge.

We observed that some buds at the center were shorter than a pair near the edge. These buds appeared to be imperfect or hindered to grow. Typical examples are shown in Fig. 4(e) representing skin lengths of 1394 *μ*m and 1158 *μ*m, as depicted by the arrows. Notably, in the simulations, these skin lengths, 1394 *μ*m and 1158 *μ*m, correspond to approximately the 8.0 and 6.3 domains, respectively. These domains are close to the threshold between the threshold generating two peaks and that generating three peaks and to the threshold between the threshold generating three peaks and that generating four peaks; this results in slow formation of the center peaks such that the center peaks at 10 time units are small or blunt, while the other peaks showed steady formation. Such slow activator peak formation may be related to the formation of short buds.

As a fundamental aspect of the reaction diffusion model, a center bud present between edge buds is located in the middle on three-bud skins. The pair of center-edge bud distances is shown in Fig. 4(f) where the *x*-axis in the plot represents the shorter distances. The skin length was 1100–1500 *μ*m. The dotted line is at an angle of 45° to the X-axis, indicating the middle. The center buds were located approximately at the center. The slight displacement from the central dotted line as the bud-bud distance increased from 400 to 600 *μ*m may again be related to slow dull activator peak formation at 10 time units on the 8.0 domain, close to the threshold generating four peaks in the numerical simulation.

Finally, we examined bud formation on short skins. The probability of bud formation on skins shorter than 700 *μ*m on day 1 was 22% (n = 4/18). Several skins showed a projection due to mesenchymal thickening on day 2 but did not grow further. This may be because of the high curvature of the shorter skin and/or the blunt peak of the activator predicted on the 3.5 domain. Since a high degree of manual handling is required to generate bioengineered skins that were less than a millimeter in thickness/length, other factors may limit bud formation, particularly on short skins.

Although the skin curvature may prevent formation of feather buds, the distances determined from the experimental observations were fairly accurately recapitulated by the simulation results of our simple model. The bud-bud distances were 416 ± 89, 418 ± 88, and 406 ± 113 *μ*m for two-bud, three-bud, and four-bud skins, respectively. The standard deviation for four-bud skin was the highest. This value increased for five-bud skins because longer skins >2000 *μ*m bend locally at a few sites.

The skin length generally increased during the 72 h of incubation as summarized in Fig. SI3 (supplementary material). Figure SI3(d) (supplementary material) shows the time course of the skin length. On average, the skin lengths were longer in samples with more bud skins. The average skin length extensions from days 1 to 2 were 2.5%, 12%, 16%, and 28% for one-, two-, three- and four-buds, respectively, while from days 2 to 3 were 2.4%, 2.6%, 6.0%, and 5.0% for one-, two-, three-, and four-buds, respectively. By visualizing gene expression, the bud pattern was minimally detectable as early as day 1.5 without projections.^25^ Our simulation may be improved by taking the skin length extension into account.^68^ However, a ratio of less than 6% would not change the results significantly. The 2-bud skins were either nearly 900 or 1200 *μ*m on day 3. The nearly 900 *μ*m skins on day 3 showed minimal length changes from days 2 to 3. In contrast, the 1200 *μ*m skins extended in length mostly with either high curvature skin. Mesenchymal cell proliferation and condensation on a free-standing mesh deformed the tissue shape in a similar *in vitro* assay using ESCs.^69^ To simulate this deformation, 3D^37^ simulations with the third component describing cell density under the influence of proliferation,^70^ chemotaxis,^47,71,72^ elastic bulk stress tensor,^73^ and surface tension^74,75^ are expected to be performed. Our numerical simulation did not contain a third component for cell density and the domain size was fixed, restricting the morphological changes of the skin tissue.

In this study, we conducted both *in vitro* experiments and numerical simulations to examine the self-organizing process of feather bud formation on bioengineered skin samples on which three or fewer buds can form. As a simple two-component Turing model with an atypical BC, mixed, or partial-flux BC, this system predicts preferred bud formation sites as approximately 280 *μ*m from each skin edge. These results also indicated the fragility of the third bud between a pair of primary buds under the influence of mechanical stress such as the change in the skin curvature.^31^ In addition, we confirmed that morphogens inducing directionality of the feather bud tilting, reflecting AP asymmetry, exist inside a native epithelium because the bud tilt disappeared in our bioengineered skin samples after reassembling single epithelial cells.

Our results revealed the high sensitivity to the size and shape, particularly the edge of bioengineered tissue composed of uniformly distributed homogeneous cells, for regenerative medicine as similar edge effects were observed recently using human embryonic stem cells.^39,40^ Such bioengineered tissue samples may provide ideal templates for studying and modeling self-organized processes as biophysical models, such as limbs, organs, hair, and teeth.^76^ Such studies would likely lead to advancements in regenerative medicine.

## METHODS

### Animals

Fertilized chicken eggs (Yamagishism, Mie, Japan) were incubated at 37.5 °C and staged according to Hamburger and Hamilton.^77^ All animal experiments were approved by the Life Science Committee of Aoyama Gakuin University (Approval ID No. A8). All *in vivo* experiments were carried out in accordance with the Guide for the Care and Use of Laboratory Animals (NIH, Bethesda, MD, USA).

### Generation and cultivation of bioengineered skin and feather buds

The details of reconstruction assays of an epithelium and mesenchyme from dissociated single cells have been described elsewhere.^25^ Briefly [schematically shown in Fig. 1(a)], embryonic dorsal skin samples were dissected from an E7 (embryonic day 7) (Hamburger and Hamilton 29) chick and treated with 1000 protease U/mL Dispase I (Wako, Osaka, Japan) in Dulbecco's modified Eagle's medium/Ham's F-12 medium (DMEM/F-12) with HEPES, 4-(2-hydroxyethyl)-1-piperazineethanesulfonic acid (Wako) 10% of fetal bovine serum (Life Technologies, Carlsbad, CA, USA) at approximately 20 ± 2 °C for 30 min. The epithelial and mesenchymal tissues of native skin were separated, followed by their individual dissociation into single cells using 0.25% trypsin (Life Technologies) and 0.1% collagenase L (Nitta Gelatin, Osaka, Japan) in Dulbecco's phosphate-buffered saline (DPBS) at 37 °C for 30 min. After gentle pipetting, epithelial and mesenchymal cells were harvested by centrifugation and the supernatant was completely removed using a GELoader Tip (GE Healthcare, Little Chalfont, UK). To reconstruct the bilayer of epithelial and mesenchymal tissues as bioengineered skin, epithelial and mesenchymal cells were injected in order using gel-loading tips into a 30–*μ*l gel drop of Cellmatrix type I-A (Nitta Gelatin), forming a pair of cylindrical cell aggregates contacting each other in parallel orientation. The diameter of each cylinder was approximately 400 ± 100 *μ*m, and lengths ranged from 452 to 1280 *μ*m for three buds or fewer. The bioengineered skin, an interface layer between epithelial and mesenchymal aggregated cells, was incubated for 30 min on a cell culture insert (Corning, Inc., Corning, NY, USA) at 37 °C and then successively cultured for 4 days at 37 °C in DMEM/F-12 with GlutaMAX supplement (Life Technologies) and 30% fetal bovine serum (Life Technologies). Phase contrast images were captured using an Axio Vert.A1 microscope and an AxioCam 503 mono (Carl Zeiss, Oberkochen, Germany). As described below, during the first 48 h, the shape of the cylindrical aggregated pair in the gel drop became flat and ellipsoid, while the interface layer was bent outward along the ellipsoidal periphery, resulting in an increase in the length of the curved skin. Using the Image J software (NIH), the interface was extracted by cross-sectioning the gray scale areas of the images since the mesenchyme layer is darker than the epithelium layer, particularly in the phase contrast images from days 2 and 3; this was confirmed by a series of gene expression experiments.^25^ The authors defined the skin edge as the site where the epithelium or mesenchyme terminates. These results are analogous to those in previous reports.^24^ The lengths of 1D skin samples and bud locations were measured every 24 h.

### Mathematical model and its numerical simulation

A two-component reaction–diffusion model with an activator and an inhibitor was applied to reflect the periodic patterns of skin appendages, such as feather buds.^5,36,38^ For example, this simplest Turing's model can be used to reproduce a formation sequence spanning a hexagonal pattern on a 2D domain by choosing nonhomogeneous initial conditions, a linear ridge for the activator at the center as a midline, and this arrangement initially generates a single bud array with equal spacing.^47,78,79^

The following are two typical equations of the model:

$$\frac{\partial A}{\partial t}=D_{A}\nabla^{2}A+\frac{p_{A}A}{1+k_{1}A^{2}}\frac{1}{1+k_{2}I}+\sigma_{A}-d_{A}A,$$ (1)

$$\frac{\partial I}{\partial t}=D_{I}\nabla^{2}I+\frac{p_{I}A^{2}}{\left(1+k_{3}A^{2}\right)}+\sigma_{I}-d_{I}I.$$ (2)

Here, *A* and *I* represent the activator and inhibitor concentrations, respectively. *D_A_* and *D_I_* are the diffusion constants, whereas *d_A_* and *d_I_* are the decay rates for the activator and the inhibitor, respectively; *p_A_* and *p_I_* are their basic production rates. The parametric reaction terms *k_1_*, *k_2_*, and *k_3_* are constants. The simulation domains were rectangles ranging from 3 × 3 to 3 × 8 length units, whose aspect ratios were similar to those of our bioengineered skin samples. The edges of the rectangular domains were rounded to mimic the skin shape as seen in the example of a 3 × 6 domain in Fig. 3(a). Initial conditions of the activator and the inhibitor were uniform with small added fluctuations (maximum 5%) throughout the domain area, assuming a uniform distribution of identical cells. Various BCs, including Neumann, mixed, and partial-flux BCs, were tested to generate activator maxima recapitulating our experimentally observed bud locations. The simulation was conducted for 30 time units until the patterns of both the activator and inhibitor concentrations reached final steady states. Numerical calculations were performed using the commercial finite element software COMSOL Multiphysics 4.4. All parameters are specified in supplementary material Table 1.

## SUPPLEMENTARY MATERIAL

See supplementary material for additional figures and the parameters of our numerical simulations.
